# Association between body mass index reduction and epicardial adipose tissue changes across pharmacological and lifestyle interventions: a multilevel meta-regression analysis

**DOI:** 10.1186/s12933-026-03299-6

**Published:** 2026-07-19

**Authors:** Aleksandra Mączyńska, Piotr Olbryś, Mateusz Smoliński, Miłosz Rosa, Marta Kacperska, Milena Froń, Paweł Leśniewski, Oliwia Łuniewska, Adam Sander, Maciej Mączewski, Hanna Czerwińska, Aleksandra Paterek, Michał Mączewski

**Affiliations:** 1 Department of Clinical Physiology, Centre of Postgraduate Medical Education, Warsaw, Poland; 2 Doctoral School at the Centre of Postgraduate Medical Education, Warsaw, Poland; 3 Student’s Cardiovascular Scientific Club “Kardioplegia”, Medical University of Warsaw, Warsaw, Poland

**Keywords:** Metaanalysis, Epicardial adipose tissue, Body mass index reduction, Cardiometabolic interventions, GLP-1 receptor agonists

## Abstract

**Background:**

Epicardial adipose tissue (EAT) is a metabolically active visceral fat depot implicated in cardiometabolic disease. Although EAT reductions are commonly attributed to weight loss, the extent to which EAT change is explained by body mass index (BMI) reduction versus intervention-specific effects remains unclear.

**Methods:**

We performed a systematic review and multilevel meta-regression of interventional studies reporting pre- and post-intervention changes in EAT and BMI. MEDLINE, Embase, and Cochrane CENTRAL were searched from inception to April 2026. Standardized mean change using pre-test standardization (SMCR) was calculated for both outcomes. Multilevel meta-regression assessed the association between BMI and EAT change while adjusting for intervention class, follow-up duration, and imaging modality. BMI-adjusted residual analyses were used descriptively to evaluate deviations from model-predicted EAT responses.

**Results:**

Forty-two studies comprising 50 intervention arms and 1,890 participants were included. Across all interventions, both EAT and BMI decreased significantly. In the multivariable meta-regression model, BMI reduction was associated with EAT reduction (β = 0.19, *p* = 0.03), although BMI explained only a limited proportion of variability in EAT response. Intervention class remained a significant moderator of EAT change (*p* < 0.001). Larger observed reductions in EAT were seen with dietary interventions, GLP-1 receptor agonists, and bariatric surgery. In descriptive residual analyses, dietary interventions and GLP-1 receptor agonists demonstrated greater-than-predicted reductions in EAT relative to the overall study-level BMI–EAT relationship. These findings should be interpreted cautiously because intervention-specific comparisons were exploratory and do not constitute formal comparative efficacy estimates.

**Discussion:**

EAT reduction is only partially explained by systemic weight loss, and substantial heterogeneity exists across intervention classes. These findings support the concept that EAT remodeling may be influenced by factors beyond generalized adiposity reduction. However, intervention-specific patterns observed in this study are descriptive and hypothesis-generating and require confirmation in adequately powered comparative studies. Moreover, overall certainty of evidence was low for pooled EAT reduction and very low for intervention-specific comparative patterns.

**Trial registration:**

PROSPERO (CRD420261367140).

**Graphical Abstract:**

**Figure:**
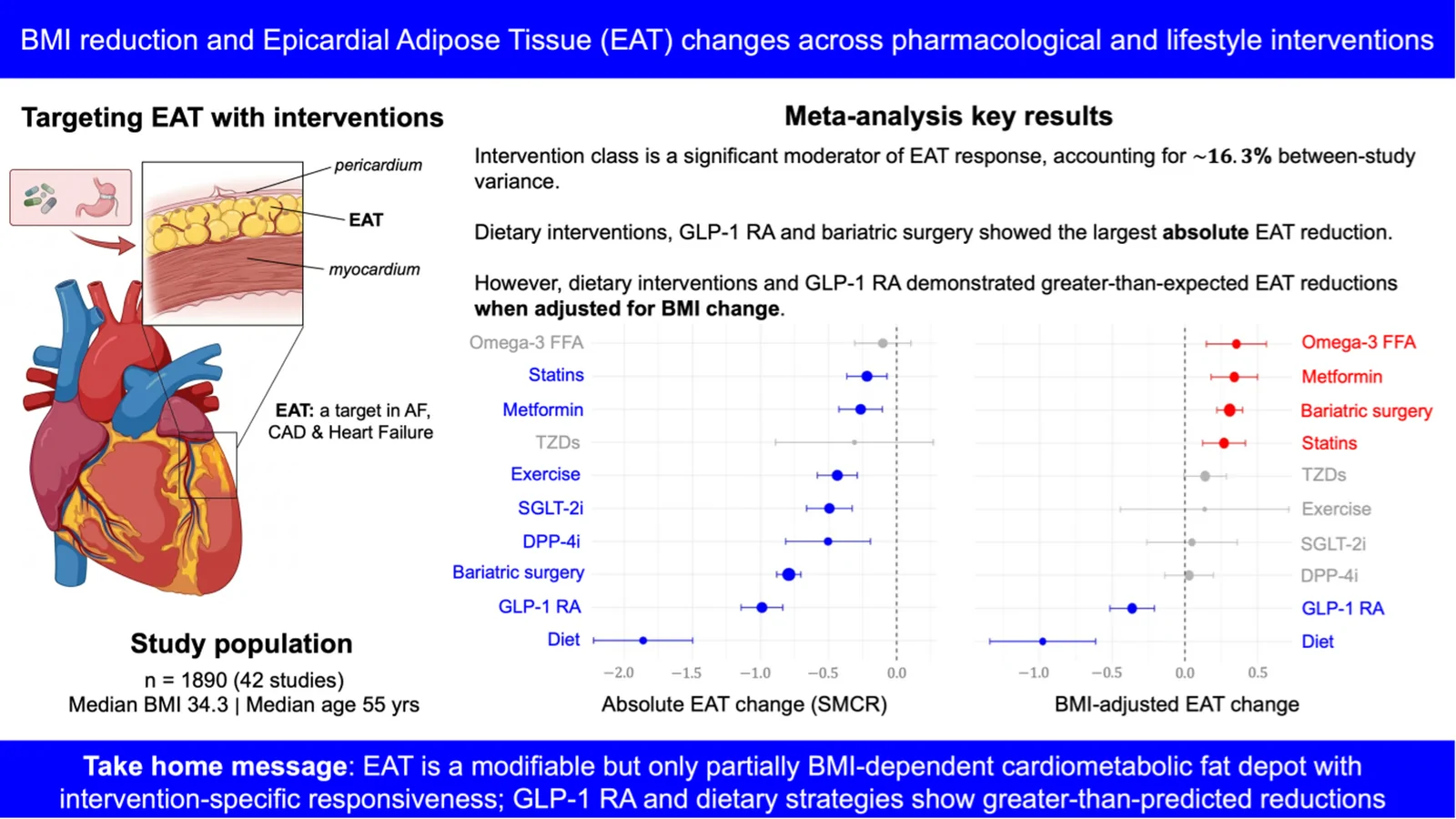

**Supplementary Information:**

The online version contains supplementary material available at https://doi.org/10.1186/s12933-026-03299-6.

## Research insights

What is currently known about this topic?

EAT is linked to obesity, diabetes, and cardiovascular disease.

Weight loss interventions generally reduce EAT quantity.

The role of BMI reduction in EAT remodeling remains uncertain.

What is the key research question?

Does EAT reduction depend mainly on BMI loss or intervention type?

What is new?

BMI explained only a modest proportion of EAT change.

Intervention class explained more EAT variability than BMI change.

Diet and GLP-1 therapies showed greater-than-predicted EAT reduction.

How might this study influence clinical practice?

EAT may serve as a complementary therapeutic and imaging target.

## Introduction

Epicardial adipose tissue (EAT) is a visceral fat depot located between the myocardium and the visceral pericardium [1]. Owing to the absence of a fascial barrier separating it from the myocardium, EAT engages in direct signaling with adjacent cardiac tissue, positioning it as a potential mediator linking systemic metabolism with cardiac structure and function through paracrine and vasocrine signaling [2]. Emerging evidence suggests that EAT volume and composition serve not only as markers of cardiovascular risk [3, 4], but also undergo pathological, pro-inflammatory and pro-fibrotic remodeling in conditions such as atrial fibrillation, coronary artery disease, and heart failure [5], highlighting its potential as a therapeutic target.

EAT can be quantified using several imaging modalities. Echocardiography measures only EAT thickness, typically over the right ventricle, whereas computed tomography (CT) provides detailed information on EAT volume, distribution, and density. Magnetic resonance imaging (MRI) also enables volumetric assessment of EAT [6].

Importantly, reductions in EAT in interventional studies have been associated with improvements in both systolic and diastolic function in healthy individuals and in patients with heart failure with preserved ejection fraction, particularly among those with obesity [7]. These findings support the concept that EAT may represent an imaging biomarker and a modifiable contributor to cardiac dysfunction and underscore the need to identify interventions that specifically target this depot.

However, EAT is strongly correlated with overall adiposity [2]. Lifestyle and pharmacological interventions that reduce body mass index (BMI) consistently decrease EAT volume, raising a key unresolved question: to what extent are reductions in EAT driven by generalized weight loss versus intervention-specific, tissue-level effects mediated by distinct metabolic pathways? Clarifying this distinction is essential for interpreting prior studies and for guiding the development of targeted therapies. Previous meta-analyses have primarily quantified the magnitude of EAT reduction following individual interventions [8, 9]. In contrast, the present study sought to examine the study-level relationship between changes in systemic adiposity and EAT across diverse intervention classes using a multilevel meta-regression framework. This approach allows assessment of the extent to which EAT remodeling parallels, or diverges from, concurrent changes in body mass index.

To address this gap, we performed a comprehensive meta-analysis and multilevel meta-regression of interventional studies to quantify the association between changes in BMI and EAT, evaluate whether this association differs across intervention classes, and evaluate EAT responses relative to BMI change.

## Methods

### Study design and reporting standards

This systematic review and meta-analysis was conducted according to PRISMA 2020 guidelines [10]. The protocol was registered on PROSPERO (CRD420261367140). The analysis was conducted as a multilevel meta-regression of longitudinal intervention studies evaluating changes in epicardial adipose tissue (EAT) quantity and body mass index (BMI).

The analytical framework explicitly modelled paired within-study changes in EAT and BMI while accounting for between-study heterogeneity and intervention-specific effects. A full analytic pipeline was implemented in R (version ≥ 4.3) using the *metafor* package. Statistical significance was defined as a two-sided p value < 0.05.

### Data sources and study selection

A systematic search of MEDLINE (PubMed), Embase, and Cochrane CENTRAL was performed from inception to 22 April 2026. Additional sources included trial registries and manual reference screening. Eligible studies met the following inclusion criteria: (1) Human interventional studies with ≥ 2 months follow-up (a minimum follow-up duration of 2 months was selected because shorter intervals are unlikely to capture stable remodeling of epicardial adipose tissue and have been excluded in several previous EAT intervention studies), (2) Reported pre- and post-intervention means and variance for both EAT and BMI, (3) Included at least one of the interventions specified in Sect.  2.8, (4) EAT quantity was assessed using echocardiography (thickness measurement), computed tomography (CT), or magnetic resonance imaging (MRI) (volume measurement). Exclusion criteria included missing variance data or non-extractable standardized mean change using pre-test standardization (SMCR) inputs for either EAT or BMI.

**Fig. 1:**
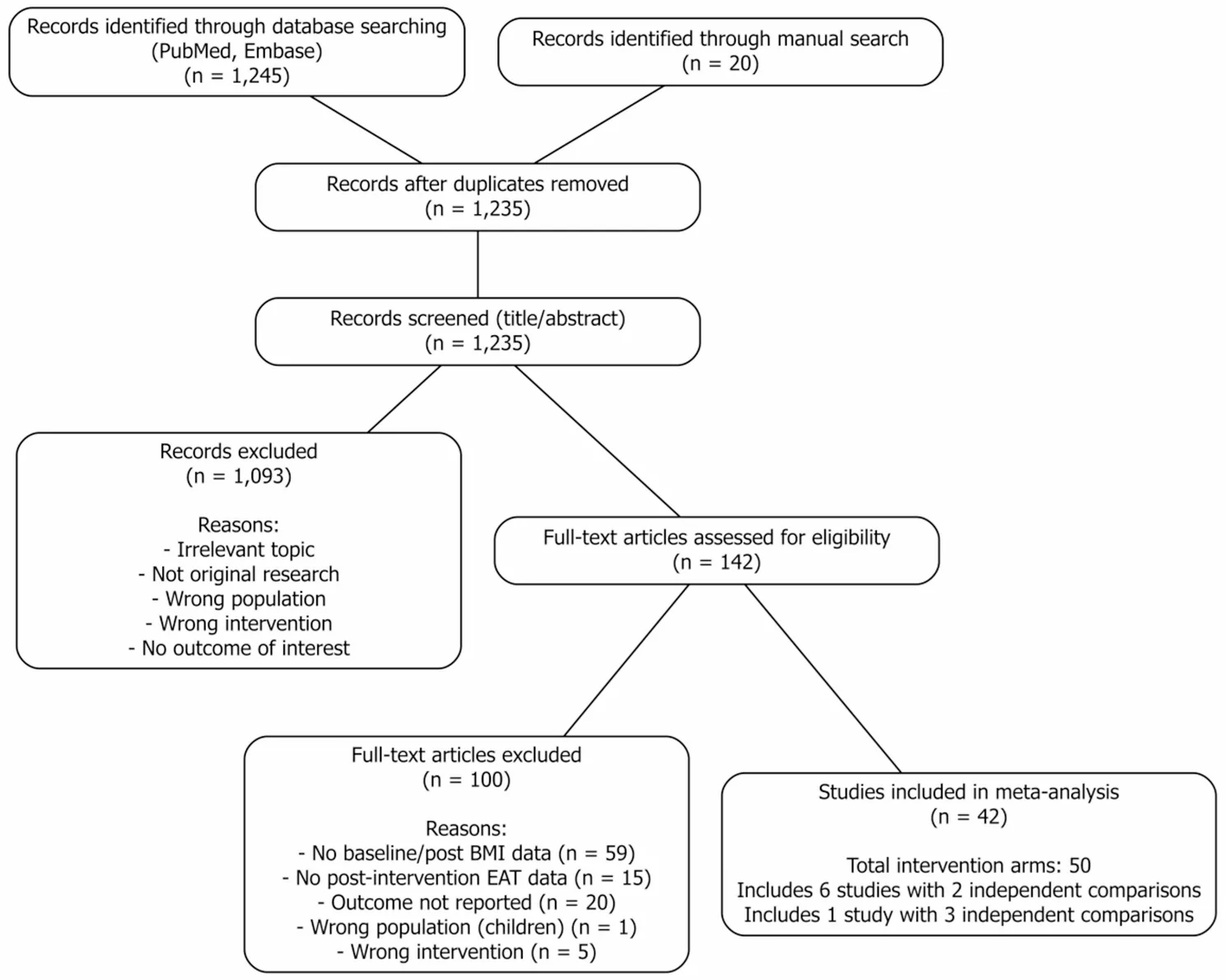
PRISMA flow diagram detailing the process that led to final study inclusion for review. Flowchart illustrating the screening of studies through title and abstract review, screening, and inclusion. *n* number of studies

Figure 1 presents the PRISMA flow diagram, illustrating the process that led to the final selection of studies for review.

**Fig. 2:**
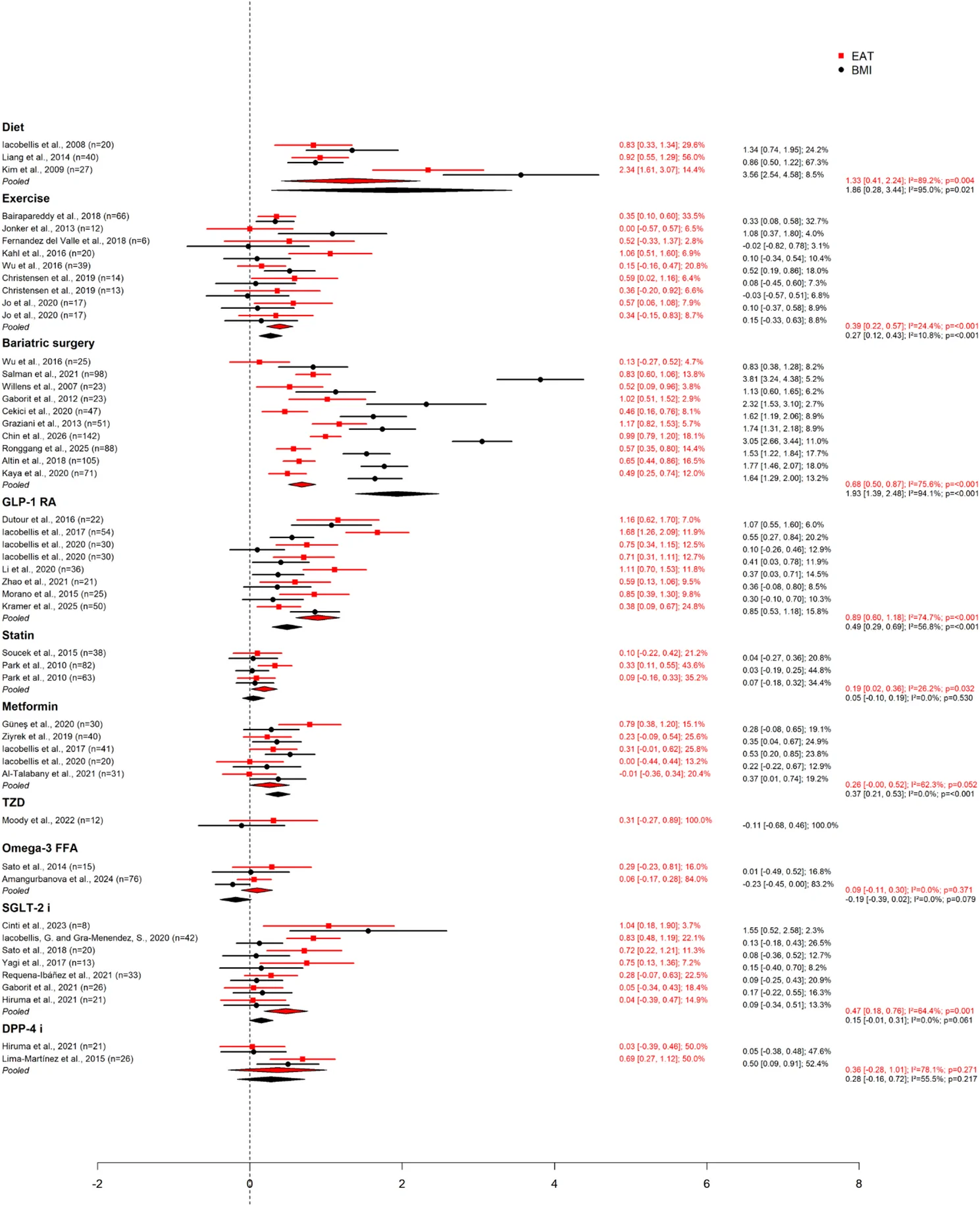
Meta-analysis of non-pharmacological and pharmacological interventions affecting epicardial adipose tissue and body mass index. Effects of non-pharmacological interventions: dietary interventions, structured exercise programs, bariatric surgery, and pharmacological interventions: glucagon-like peptide 1 receptor agonists (GLP-1 RA), metformin, statins, dipeptidyl peptidase-4 inhibitors (DPP-4i), omega-3 fatty acids, sodium-glucose cotransporter-2 inhibitors (SGLT-2i), and thiazolidinediones (TZD) on epicardial adipose tissue (EAT) measures (thickness or volume) and body mass index (BMI). Standardized mean change using pre-test standardization (SMCR) with confidence intervals (CI) and weight is shown for each study and for the pooled intervention groups. Heterogeneity (I²) and p value is shown for each pooled intervention group. Red - EAT, black - BMI

### Outcomes

The primary outcome was change in EAT quantity. The secondary outcome was change in BMI. A secondary analytical objective was to quantify the association between changes in BMI and changes in EAT, interpreted as the coupling between systemic and regional adiposity responses, and to evaluate whether this relationship was modified by intervention type (FIg. 2).

### Data extraction

Data were independently extracted by two reviewers, including study design, study population characteristics, sample size, intervention type, intervention class, follow-up duration, imaging modality, and pre/post means and standard deviations for EAT and BMI.

### Handling of multi-arm studies

Several studies contributed more than one intervention arm. To account for the resulting dependency structure, intervention arms were modeled as nested within studies using a multilevel random-effects framework (random = ~ 1 | study/arm). This approach explicitly partitions variance into between-study and between-arm components while avoiding double counting of studies contributing multiple intervention groups. Because effect sizes were calculated from within-arm pre–post changes, no shared comparator groups contributed to multiple effect estimates.

### Effect size calculation

Effect sizes were computed as SMCR for each studyarm:

$$\:{y}_{EAT,i}=\frac{EA{T}_{post}-\:EA{T}_{pre}}{S{D}_{pre}}\:$$

$$\:{y}_{BMI,i}=\frac{BM{I}_{post}-\:BM{I}_{pre}}{S{D}_{pre}}$$

Study-level effect sizes were calculated using established methods for paired pre–post designs [11], assuming a within-subject correlation of *r* = 0.5. This assumption was tested in sensitivity analyses (*r* = 0.3–0.7). Effect size computation and variance estimation were implemented using the escalc() function in the metafor package.

### Primary multilevel meta-regression model

The primary model evaluated the association between BMI change and EAT change while accounting for intervention class, follow-up duration, and imaging modality using a multilevel meta-regression framework:

$$ \begin{aligned} \:y_{{EAT,i}} = & \:\beta \:_{0} + \:\beta \:_{1} y_{{BMI,i}} + \:\sum \: _{k} \gamma \:_{k} Group_{k} \\ & + \beta \:_{2} Duration_{i} + \sum \: _{k} d_{m} Imaging_{m} + u_{{study}} + u_{{arm}} + \:\varepsilon \:_{i} \\ \end{aligned} $$

where:

$$\:{u}_{study}\sim\:N\left(0,\:{\tau\:}^{2}study\right)$$

$$\:{u}_{arm}\sim\:N\left(0,\:{\tau\:}^{2}arm\right){\epsilon\:}_{i}\sim\:N\left(0,\:{v}_{i}\right)$$

Intervention class (γk) was included as a covariate to adjust for systematic differences between intervention types and was not interpreted as a comparative ranking framework. Models were estimated using restricted maximum likelihood (REML) via rma.mv.

### BMI-adjusted residual variation in EAT (descriptive analysis)

To quantify deviations in EAT change not explained by concurrent BMI changes, study-level residuals were derived from the fitted multilevel meta-regression model:

$$\:{e}_{i}=\:{y}_{EAT,i}-{\widehat{y}}_{EAT,i}$$

where $\:{\widehat{y}}_{EAT,i\:}$represents BMI- and covariate-adjusted predicted EAT change from the multilevel model.

These residuals represent the difference between observed and BMI-predicted EAT responses and are therefore interpreted as descriptive deviations from the expected EAT–BMI relationship, rather than estimates of causal or BMI-adjusted effects.

**Fig. 3:**
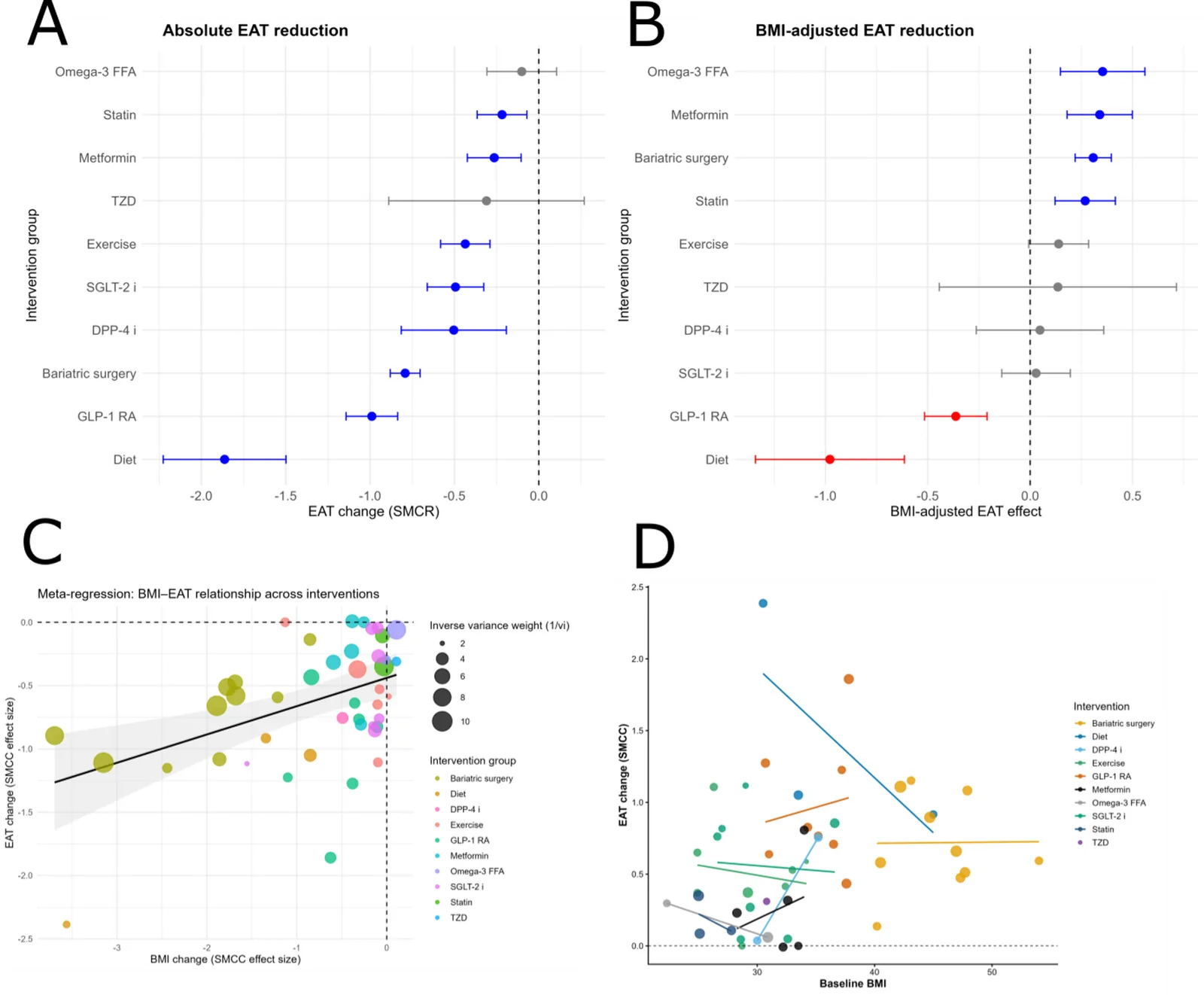
Epicardial adipose tissue response to cardiometabolic interventions: magnitude, BMI coupling, and baseline modifiers. **A** Pooled standardized mean change scores (SMCR) for epicardial adipose tissue (EAT) across intervention groups. Effect sizes are weighted by inverse variance; positive values indicate greater reduction in EAT quantity. Interventions with statistically significant within-group changes (*p* < 0.05) are shown in blue, while non-significant interventions are shown in grey. The ordering shown does not represent comparative efficacy and should not be interpreted as evidence of superiority between interventions. **B** Residualized EAT effects after adjustment for BMI change using meta-regression. Values represent deviation of observed EAT change from that predicted by BMI change (i.e., BMI-adjusted improvement in EAT). Interventions with significantly greater-than-predicted improvement (Diet and GLP-1 receptor agonists) are shown in blue. Non-significant interventions (including Exercise, TZD, DPP-4 inhibitors, and SGLT-2 inhibitors) are shown in grey, while interventions with significantly worse-than-predicted outcomes are shown in red. Positive values indicate worse-than-expected EAT reduction relative to BMI change, whereas negative values indicate better-than-expected outcomes. **C** Meta-regression EAT vs. BMI change. Each point represents an intervention effect estimate, weighted by study sample size (bubble size proportional to √n). The solid line shows the fitted linear meta-regression between standardized mean change in BMI and standardized mean change in EAT, with 95% confidence interval (shaded area). The dashed horizontal and vertical lines indicate no change in EAT and BMI, respectively. Interventions located below the regression line demonstrate greater-than-expected reduction in EAT relative to BMI change, suggesting potential BMI-adjusted or EAT-specific effects. Interventions above the line indicate lower-than-expected EAT reduction given BMI change, consistent with predominantly weight- mediated or dissociated effects on epicardial fat. **D** Association between baseline BMI and EAT response across interventions. Each point represents an individual study estimate of standardized mean change in EAT, plotted against baseline BMI, with symbol size proportional to study sample size. Colored lines represent intervention-specific linear regression fits. The horizontal dashed line indicates no change in EAT

For visualization, residuals were summarized within intervention classes using inverse-variance weighting. These summaries are presented in Fig. 3 to illustrate patterns of over- or under-performance relative to BMI-predicted EAT change.

All residual analyses were performed post hoc for visualization of model fit and heterogeneity and were not used to generate independent effect estimates.

To facilitate interpretation of intervention effects, intervention classes ordered descriptively according to two complementary metrics:

1. Raw EAT change (SMCR): Pooled standardized mean change in EAT within each intervention class, calculated using inverse-variance weighting of study-level SMCR estimates.
2. BMI-adjusted EAT deviation (residual SMCR): Pooled residual EAT change derived from the multilevel meta-regression model, representing deviation of observed EAT change from BMI-predicted change.

### Intervention classification

All analyses used the predefined **Group variable**, including:

- Diet.
- Exercise.
- Bariatric surgery.
- GLP-1 RAs.
- SGLT-2 inhibitors.
- Metformin.
- Statins.
- DPP-4 inhibitors.
- Omega-3 fatty acids.
- Thiazolidinediones.

Interventions were classified according to the primary intended therapeutic strategy. Where exercise or lifestyle counseling accompanied pharmacological therapy, the intervention was categorized according to the dominant intervention specified by study investigators.

Because intervention classes differed substantially in the magnitude of BMI change achieved, BMI-adjusted residual analyses may be influenced by the underlying distribution of weight loss across interventions. Consequently, intervention-specific deviations should be interpreted as descriptive departures from the average study-level BMI–EAT relationship rather than evidence of preferential EAT mobilization.

### Heterogeneity

Between-study heterogeneity was assessed using τ², I², and Cochran’s Q. Explained heterogeneity was calculated as:

$$\:{R}^{2}=\:\frac{{\tau\:}_{null}^{2}-\:{\tau\:}_{model}^{2}}{{\tau\:}_{null}^{2}}\:\:$$

where τ² values were compared between nested models.

Variance decomposition was performed using the variance components estimated from the multilevel random-effects model. The proportion of total variance attributable to between-study, between-arm (within-study), and sampling variance components was calculated by dividing each variance component by the total estimated variance. These proportions were used descriptively to characterize the hierarchical structure of heterogeneity.

### Sensitivity analyses

Robustness was evaluated using:

- variation in assumed correlation (*r* = 0.3–0.7).
- exclusion of small studies (*n* < 20).
- leave-one-out diagnostics.
- stratification by imaging modality.

### Risk of bias (study-level) and certainty

Risk of bias was assessed at the study level using a predefined study-quality framework informed by selected methodological principles from ROBINS-I and aligned with the risk-of-bias domain of the GRADE approach. The assessment focused on four design-related characteristics considered particularly relevant to the included studies (Table 1): comparator type (placebo, active comparator, usual care, or none), randomization (yes/no), study design (controlled vs. single-arm), and sample size. Studies were classified as low risk of bias if they were randomized controlled trials with an appropriate comparator and a sample size ≥ 20. Moderate risk of bias was assigned to randomized studies with small sample sizes (< 20) or non-randomized but controlled studies. High risk of bias was assigned to non-randomized single-arm studies, studies without a comparator, or studies with very small sample sizes (< 20) in combination with other design limitations.

**Table 1:** Characteristics of studies included in the meta-analysis evaluating the effects of lifestyle, surgical, and pharmacological interventions on cardiac adipose tissue assessed by different imaging modalities

Intervention	Study	Comparator	Study structure		Imaging modality	Population	Baseline BMI (kg/m^2^)	*N*	Intervention subtype	Intervention duration (months)	Risk of bias	Relative magnitude of change:	
			Randomization	Design								EAT	BMI
EXERCISE	Bairapareddy et al. [17]	P	Y	C	Echo	Obesity	29.2	66	Aerobic exercise	3	L	0.13	0.03
	Jonker et al. [18]	N	N	S	MRI	Diabetes	28.7	12	Aerobic exercise	7	H	0.00	0.05
	Fernandez del Valle et al. [19]	P	Y	C	MRI	Obesity	34.2	6	High-intensity, moderate-volume muscular endurance resistance training	2	H	0.16	0.00
	Kahl et al. [20]	U	Y	C	MRI	Major depression	26.3	20	Aerobic exercise	2	M	0.12	0.02
	Wu et al. [21]	A	N	C	CT	Obesity	32.6	39	Aerobic exercise	3	M	0.06	0.07
	Christensen et al. [22]	A	Y	C	MRI	Obesity	33.0	14	Endurance training	3	M	0.26	0.01
	Christensen et al. [22]	A	Y	C	MRI	Obesity	32.4	13	Resistance training	3	M	0.18	0.00
	Jo et al. [23]	A	Y	C	Echo	Hypertension and metabolic syndrome	24.9	17	High-intensity interval training	2	M	0.20	0.01
	Jo et al. [23]	A	Y	C	Echo	Hypertension and metabolic syndrome	24.9	17	Moderate-intensity interval training	2	M	0.11	0.02
DIET	Iacobellis et al. [24]	N	N	S	Echo	Obesity	45.0	20	Low caloric diet (900 kcal)	6	H	0.33	0.16
	Liang et al. [25]	N	N	S	MRI	Obesity	33.5	40	Low caloric diet (1200) + mild exercise program	3	H	0.32	0.11
	Kim et al. [26]	N	N	S	Echo	Obesity	30.5	27	Low caloric diet (reduction by 27% to 1546 kcal)	3	H	0.17	0.11
BARIATRIC SURGERY	Wu et al. [21]	A	N	C	CT	Obesity	40.2	25		3	M	0.05	0.15
	Salman et al. [27]	N	N	S	Echo	Obesity	44.7	98		12	H	0.13	0.28
	Willens et al. [28]	N	N	S	Echo	Obesity	54.0	23		8	H	0.25	0.26
	Gaborit et al. [29]	N	N	S	MRI	Obesity	43.1	23		6	H	0.28	0.25
	Cekici et al. [30]	N	N	S	Echo	Obesity	47.3	47		6	H	0.10	0.21
	Graziani et al. [31]	U	N	C	Echo	Obesity	47.9	51		24	M	0.31	0.25
	Chin et al. [7]	N	N	S	Echo	Obesity	42.2	142		12	H	0.31	0.33
	Ronggang et al. [32]	N	N	S	CT	Obesity	40.5	88		11	H	0.08	0.26
	Altin et al., 2018 [33]	N	N	S	Echo	Obesity	47.0	105		6	H	0.15	0.29
	Kaya et al. [34]	N	N	S	Echo	Obesity	47.7	71		6	H	0.11	0.23
STATIN	Soucek et al. [35]	P	Y	C	CT	AF	27.8	38	Atorvastatin	3	L	0.06	0.01
	Park et al. [36]	A	N	C	Echo	CAD	25.0	82	Atorvastatin	7	M	0.11	0.00
	Park et al. [36]	A	N	C	Echo	CAD	25.1	63	Simvastatin + ezetimibe	7	M	0.03	0.01
GLP-1 RA	Dutour et al., 2016 [37]	U	Y	C	MRI	Diabetes	37.2	22	Exenatide	6	L	0.09	0.05
	Iacobellis et al. [38]	A	Y	C	Echo	Diabetes and obesity	37.8	54	Liraglutide	6	L	0.35	0.11
	Iacobellis et al. [39]	A	N	C	Echo	Diabetes and obesity	34.3	30	Semaglutide	3	M	0.21	0.01
	Iacobellis et al. [39]	A	N	C	Echo	Diabetes and obesity	36.5	30	Dulaglutide	3	M	0.17	0.07
	Li et al. [40]	N	N	S	Echo	Diabetes and obesity	30.7	36	Liraglutide	3	H	0.30	0.06
	Zhao et al. [41]	N	N	S	MRI	Diabetes and obesity	31.0	21	Liraglutide	3	H	0.21	0.05
	Morano et al. [42]	A	N	C	Echo	Diabetes	35.2	25	Liraglutide/exenatide	3	M	0.15	0.04
	Kramer et al. [43]	P	Y	C	MRI	HFpEF and obesity	37.6	50	Tirzapatide	12	L	0.26	0.12
METFORMIN	Güneş et al. [44]	N	N	S	Echo	Obesity	34.0	30	Metformin	3	H	0.27	0.06
	Ziyrek et al. [45]	N	N	S	Echo	Diabetes	28.3	40	Metformin	3	H	0.06	0.03
	Iacobellis et al., 2017 [38]	A	Y	C	Echo	Diabetes and obesity	32.6	41	Metformin	6	L	0.07	0.11
	Iacobellis et al. [39]	A	N	C	Echo	Diabetes and obesity	33.5	20	Metformin	3	M	0.00	0.04
	Al-Talabany et al. [46]	A	Y	C	MRI	Obesity	32.2	31	Metformin	12	L	0.00	0.04
TZD	Moody et al. [47]	N	N	S	MRI	Diabetes	30.8	12	Pioglitazone	6	H	0.08	-0.02
OMEGA-3 FFA	Sato et al. [48]	U	Y	C	CT	CAD	22.3	15	EPA	6	M	0.09	0.00
	Amangurbanova et al. [49]	N	Y	C	CT	CAD	30.9	76	EPA + DHA	30	M	0.03	-0.03
SGLT-2 i	Cinti et al. [50]	P	Y	C	Echo	Diabetes and CAD	29.0	8	Dapagliflozin 10 mg	2	H	0.19	0.01
	Iacobellis and Gra-Menendez [51]	P	Y	C	Echo	Diabetes and obesity	36.6	42	Dapagliflozin 10 mg	6	L	0.20	0.03
	Sato et al. [52]	U	Y	C	CT	Diabetes and CAD	26.6	20	Dapagliflozin 10 mg	6	M	0.14	0.02
	Yagi et al. [53]	N	N	S	Echo	Diabetes	27.0	13	Canagliflozin	6	H	0.22	0.03
	Requena-Ibáñez et al. [54]	P	Y	C	MRI	HFrEF	29.4	33	Empagliflozin	6	L	0.04	0.02
	Gaborit et al. [55]	P	Y	C	MRI	Diabetes	32.6	26	Empagliflozin	4	L	0.01	0.02
	Hiruma et al. [56]	A	Y	C	MRI	Diabetes	28.6	21	Empagliflozin	3	M	0.02	0.02
DPP-4 i	Hiruma et al. [56]	A	Y	C	MRI	Diabetes	30.0	21	Sitagliptin	3	M	0.01	0.01
	Lima-Martínez et al. [57]	N	N	S	Echo	Diabetes	35.2	26	Sitagliptin + metformin	6	H	0.19	0.09

Studies are categorized by intervention type and include details on imaging modality, population, sample size, intervention subtype, duration (months), and measurement units. Cardiac adipose tissue was quantified using echocardiography (linear thickness, mm), computed tomography, or magnetic resonance imaging (volumetric or mass measurements, ml or g). AF – atrial fibrillation; BMI – body mass index; CAD – coronary artery disease; CT – computed tomography; DPP-4 i – dipeptidyl peptidase-4 inhibitor; Echo – echocardiography; EPA – eicosapentaenoic acid; DHA – docosahexaenoic acid; FFA – free fatty acids; GLP-1 RA – glucagon-like peptide-1 receptor agonist; HFpEF – heart failure with preserved ejection fraction; HFrEF – heart failure with reduced ejection fraction; MRI – magnetic resonance imaging; N – sample size; SGLT-2 i – sodium–glucose cotransporter-2 inhibitor; TZD – thiazolidinedione. Comparator: A - active; N - none; P - placebo; U - usual care. Study structure: Randomization: N - no; Y - yes. Design: C - controlled; S - single arm. Risk of bias was assessed as high (H), moderate (M) or low (L). Relative magnitude of change in EAT or BMI was calculated as (pre-intervention value − post-intervention value) / pre-intervention value

This approach was not intended as a formal ROBINS-I assessment. Rather, it provided a pragmatic evaluation of key study design features to support evidence grading within the GRADE framework. Consequently, some domains included in comprehensive risk-of-bias tools, such as confounding, outcome measurement bias, and selective reporting, were not systematically assessed.

### Publication bias

Publication bias was assessed using funnel plot inspection, Egger’s regression test for funnel plot asymmetry, and Duval and Tweedie’s trim-and-fill procedure. Because several studies contributed multiple intervention arms, publication bias analyses were performed at the study level using aggregated effect estimates. The trim-and-fill method was used to estimate the potential impact of unpublished studies on the pooled effect size.

### Data availability

All derived effect sizes, cleaned datasets, and analysis scripts are available from the corresponding author upon reasonable request and can be fully reconstructed from the provided R pipeline.

## Results

### Study characteristics

The systematic search (presented in detail in the Supplementary Results and Fig. 1) identified a total of 42 eligible studies, contributing 50 independent intervention arms due to multi-arm trial designs (Table 1). Overall, 1,890 participants were included in the final analysis. Individual study sample sizes ranged from 6 to 142 participants. The included population was predominantly characterized by excess adiposity, with a median BMI of 34.3 kg/m² (interquartile range [IQR] 29.7–41.7), and a median age of 55 years (IQR 51–60): 67% of study participants had obesity, while 28% had type 2 diabetes mellitus. Common comorbidities included coronary artery disease, hypertension, atrial fibrillation, heart failure, and major depressive disorder, reflecting a metabolically high-risk clinical population.

### Overall pooled effects of interventions (descriptive models)

Across all interventions, both epicardial adipose tissue (EAT) and body mass index (BMI) decreased significantly. In the multilevel meta-analysis, the pooled standardized mean change with repeated measures (SMCR) for EAT was − 0.56 (95% CI − 0.68 to − 0.44; *p* < 0.001), and for BMI − 0.78 (95% CI − 1.07 to − 0.50; *p* < 0.001). These estimates represent marginal (unadjusted) pooled effects across intervention arms.

Variance decomposition indicated that heterogeneity in EAT effects was primarily attributable to between-arm (within-study) differences, accounting for 62.6% of total variance, followed by within-study variance (27.9%), with a smaller contribution from between-study heterogeneity (9.4%). In contrast, variability in BMI change was almost entirely driven by between-study heterogeneity (93.3%), with negligible contribution from between-arm variance and a small within-study component (6.7%) (Tab. S1).

### Multivariable meta-regression model (BMI–EAT coupling)

In the primary multivariable multilevel meta-regression model adjusting for BMI change, intervention class, follow-up duration, and imaging modality, changes in BMI were significantly associated with changes in EAT quantity (β = 0.19, *p* = 0.03). This indicates that reductions in systemic adiposity were accompanied by reductions in epicardial fat across studies.

However, the magnitude of this association was moderate, suggesting that BMI explains only part of the variability in EAT response. Importantly, this relationship remained robust after adjustment for intervention class, duration, and imaging modality.

### Effect of intervention class on EAT responses

Within the same multivariable model, intervention class remained a significant moderator of EAT change (QM test, *p* < 0.001), indicating substantial heterogeneity in treatment-related effects beyond BMI-mediated changes.

Adjustment for BMI change, follow-up duration, and imaging modality reduced residual heterogeneity, with the model explaining approximately 16.3% of between-study variance (pseudo-R² = 16.3%). Despite this reduction, substantial unexplained heterogeneity remained, indicating the presence of additional biological or methodological modifiers of EAT responsiveness.

### Intervention-specific effects (adjusted and unadjusted patterns)

Unadjusted SMCR estimates demonstrated marked heterogeneity across interventions. The largest reductions in EAT were observed with GLP-1 receptor agonists, dietary interventions, and bariatric surgery. Intermediate effects were observed for SGLT-2 inhibitors, DPP-4 inhibitors, and exercise, whereas metformin, statins, omega-3 fatty acids, and thiazolidinediones showed smaller or inconsistent effects.

In the multivariable model adjusting for BMI change and covariates, GLP-1 receptor agonists and dietary interventions remained associated with greater-than-expected reductions in EAT relative to BMI change. This pattern is consistent with, but does not establish, the possibility of adipose tissue-specific effects beyond systemic weight loss. In contrast, exercise, SGLT-2 inhibitors, and most cardiometabolic pharmacotherapies showed effects more closely aligned with BMI-predicted changes.

These findings should be considered exploratory and may reflect differences in patient populations, study design, intervention characteristics, or other unmeasured factors. These intervention-level comparisons are descriptive and reflect model-adjusted deviations rather than direct head-to-head comparisons.

### Interpretation of BMI-adjusted residuals

BMI-adjusted residuals were used solely as descriptive measures of deviation from the average study-level BMI–EAT relationship. These residuals should not be interpreted as estimates of causal, mechanistically BMI-adjusted, or intervention-specific biological effects. Rather, they reflect the combined influence of unmeasured biological, methodological, and clinical factors not captured by the multivariable model.

### BMI-adjusted deviations in EAT (residual analysis)

Residuals from the multivariable meta-regression model were derived as the difference between observed and model-predicted EAT change conditional on BMI change, intervention class, follow-up duration, and imaging modality.

These residuals demonstrated systematic variation across intervention classes. Dietary interventions and GLP-1 receptor agonists showed greater-than-predicted reductions in EAT relative to systemic weight loss and covariates. Bariatric surgery, metformin, statins, and omega-3 fatty acids showed smaller-than-expected effects, whereas SGLT-2 inhibitors, exercise, DPP-4 inhibitors, and thiazolidinediones were broadly consistent with model-predicted responses.

These residuals represent deviations from the expected multivariable BMI–EAT relationship and are descriptive measures of model fit and heterogeneity rather than BMI-adjusted or causal effects.

The relationship between BMI change and EAT change across intervention classes is shown in Fig. 3C, with study-level estimates weighted by inverse variance. These visual summaries should not be interpreted as comparative treatment rankings. Because interventions were not compared within a common randomized framework, the analyses do not provide evidence regarding relative efficacy between intervention classes.

### Effect of follow-up duration and imaging modality

Follow-up duration was not significantly associated with changes in EAT (*p* = 0.91), indicating that treatment duration within the observed range did not meaningfully modify the BMI–EAT relationship.

Imaging modality (CT, echocardiography, or MRI) was not a significant moderator of EAT change in the multivariable model, suggesting that measurement modality did not systematically bias effect estimates.

### Baseline BMI as a modifier of response (exploratory analysis)

Baseline BMI was not associated with the magnitude of EAT reduction (*p* = 0.985). No consistent interaction was observed between baseline BMI and intervention class. Although the overall interaction model reached statistical significance in exploratory analyses, individual interaction terms were not significant, suggesting that between-study heterogeneity is likely driven by multiple distributed factors rather than a single baseline characteristic.

Residual heterogeneity remained substantial, reinforcing the presence of additional unmeasured biological and methodological moderators influencing EAT responsiveness.

### Heterogeneity, model performance, and sensitivity analyses

Substantial heterogeneity remained in the final multivariable model, although reduced compared with simpler models. Intervention class was the strongest contributor to explained heterogeneity, followed by BMI change, while duration and imaging modality contributed minimally.

Sensitivity analyses confirmed robustness of findings. Variation in the assumed within-subject correlation (*r* = 0.3–0.7) did not materially alter effect estimates. Leave-one-out analyses confirmed that no single study disproportionately influenced results. Stratified analyses by imaging modality produced consistent estimates, supporting the stability of the findings across measurement techniques.

These findings are consistent with variance decomposition results, which indicate that EAT variability is predominantly driven by within-study (between-arm) differences, whereas BMI variability is largely explained by between-study heterogeneity.

### Certainty of evidence

The overall certainty of evidence for EAT reduction was rated as low, primarily due to substantial heterogeneity (I² > 70%), variability in study design, and imprecision related to small sample sizes.

The certainty of evidence for the BMI–EAT association was rated as low to very low, driven by residual heterogeneity, indirectness across intervention types, and limitations inherent to study-level meta-regression.

Across intervention classes, certainty of evidence was predominantly low to very low. Evidence for exercise and dietary interventions was rated as very low due to imprecision and risk of bias in non-randomized or single-arm studies. Among pharmacological therapies, certainty ranged from low to moderate, with SGLT-2 inhibitors showing comparatively higher certainty due to a greater proportion of randomized controlled trials.

Publication bias analyses were performed on study-level effect estimates. Visual inspection of the funnel plot suggested mild asymmetry. Egger’s regression test indicated possible small-study effects (z = 2.05, *p* = 0.040). However, trim-and-fill analysis did not identify any potentially missing studies (k0 = 0), and the pooled effect estimate remained unchanged after adjustment (SMCR 0.584, 95% CI 0.464–0.705 before and after correction). These findings suggest that potential publication bias is unlikely to have materially influenced the overall results (Tab. S5-S6).

A detailed GRADE evidence profile is provided in Supplementary Table S7. Certainty was generally low for pooled EAT reduction and very low for intervention-specific comparative patterns because of heterogeneity, indirectness, imprecision, and limitations inherent to study-level meta-regression.

## Discussion

### Principal findings

This meta-analysis provides four principal findings. First, a broad range of cardiometabolic interventions—including lifestyle, pharmacological, and surgical approaches—reduce EAT quantity in populations predominantly with obesity and diabetes. Second, the magnitude of EAT reduction varies substantially across intervention classes and is predominantly driven by within-study (between-arm) differences, rather than between-study variability and is not explained by baseline systemic adiposity, as assessed by BMI. Third, although reductions in EAT are associated with concurrent reductions in BMI, this relationship is modest, and patterns of variability differ markedly between outcomes, with EAT showing predominantly within-study variation and BMI largely reflecting between-study differences, indicating that systemic weight loss explains only a limited proportion of EAT remodeling. Fourth, certain interventions, particularly dietary strategies and GLP-1 RAs, demonstrate greater-than-expected reductions in EAT relative to BMI change, suggesting possible depot-specific or non-weight-mediated effects.

### Dissociation between systemic adiposity and epicardial fat remodeling

A central finding of this study is the partial dissociation between systemic adiposity and EAT response to intervention. While EAT is strongly correlated with measures of systemic adiposity at baseline—including BMI, visceral adipose tissue, and subcutaneous fat in prior observational studies [2]—this relationship does not appear to translate into predictable responsiveness to therapeutic interventions.

In our analysis, baseline BMI was not associated with the magnitude of EAT reduction and explained only a small fraction of between-study heterogeneity. Across studies, BMI change accounted for only a modest proportion of variance in EAT change, with the majority of variability arising at the within-study (between-arm; Level 2) level rather than between-study differences. Together, these findings indicate that systemic adiposity explains only a limited proportion of EAT remodeling and that most variability in EAT response occurs within studies across intervention arms.

Furthermore, although reductions in BMI were significantly associated with reductions in EAT, the strength of this relationship was modest. Across studies, BMI explained only a limited proportion of variance in EAT change, with substantial residual heterogeneity remaining unexplained. Together, these findings support a model in which EAT is partially, but not exclusively, regulated by systemic energy balance.

Biologically, this dissociation is consistent with the unique properties of EAT [1]. Unlike other visceral fat depots, EAT is in direct contiguity with the myocardium and lacks a fascial boundary, enabling bidirectional paracrine and vasocrine signaling [2]. This microenvironmental proximity may render EAT sensitive not only to systemic metabolic state but also to local cardiac, inflammatory, and neurohormonal signals. Such mechanisms may contribute to differential responsiveness to interventions that cannot be fully captured by changes in BMI alone.

Clinically, these findings suggest that BMI may be an incomplete surrogate for predicting changes in cardiac adiposity. This has implications for both risk stratification and therapeutic monitoring, particularly in populations where EAT is implicated in cardiac dysfunction, such as heart failure and coronary artery disease [3, 4].

### Intervention-dependent effects and biological heterogeneity

We observed substantial heterogeneity in EAT reduction across intervention classes, with nearly half of between-study variability attributable to intervention type. This indicates that EAT responsiveness is, at least in part, intervention-dependent rather than solely a function of weight loss.

#### Lifestyle interventions

Dietary interventions demonstrated consistently large reductions in EAT, both in absolute terms and relative to BMI change. One plausible explanation is that sustained caloric restriction induces a more stable and prolonged negative energy balance compared with exercise-based interventions, thereby favoring mobilization of ectopic lipid stores, including EAT.

In contrast, exercise-based interventions may induce more complex metabolic adaptations, including compensatory changes in substrate utilization and circulating free fatty acid turnover, potentially attenuating net reductions in specific adipose depots. Distinct pathways of EAT reduction with caloric restriction and exercise are supported by evidence of additive effects [12]. However, these interpretations remain speculative, and differences in study populations and baseline metabolic status may also contribute to observed variability.

Bariatric surgery demonstrated large absolute reductions in EAT, consistent with its profound effects on systemic energy balance. However, relative to BMI change, reductions in EAT were not disproportionately large, suggesting that surgical weight loss primarily reflects global adiposity reduction rather than selective depletion of cardiac fat.

#### Pharmacological interventions

Among pharmacological therapies, GLP-1 receptor agonists emerged as the class associated with the largest reductions in EAT, both in absolute terms and after adjustment for BMI change. Importantly, these findings should be interpreted cautiously, as this analysis is not based on head-to-head comparisons and is subject to heterogeneity in study design, population characteristics, and follow-up duration.

Nevertheless, several biological mechanisms may plausibly contribute to this pattern. GLP-1 receptor agonists induce potent and sustained appetite suppression, leading to reduced caloric intake and a metabolic profile that resembles dietary caloric restriction [13]. This aligns with the strong effects observed for dietary interventions, supporting the hypothesis that sustained energy deficit is a key driver of EAT reduction.

In contrast, other glucose-lowering agents exhibit different mechanistic profiles. Sodium-glucose cotransporter-2 inhibitors (SGLT2i) primarily induce urinary glucose excretion and negative energy balance without central appetite suppression. Metformin acts mainly through AMPK activation, reduced hepatic gluconeogenesis, and improved insulin sensitivity, with relatively modest effects on body weight and appetite. Statins, while reducing cardiovascular risk through lipid-lowering and anti-inflammatory effects, have limited direct impact on adipose tissue mass.

Emerging evidence suggests that GLP-1 receptors may be expressed at low levels in adipose tissue, including adipocytes, raising the possibility of direct or indirect effects on lipid metabolism and fatty acid oxidation [14]. Although these mechanisms remain incompletely characterized, they may contribute to preferential effects on metabolically active visceral depots such as EAT.

Importantly, our findings should not be interpreted as evidence of superiority of one pharmacological class over another. Rather, they highlight differential patterns of association between pharmacological mechanisms and changes in cardiac fat, warranting further investigation in controlled comparative studies.

### Clinical implications

These findings have several clinically relevant implications. First, EAT should be considered a dynamic and modifiable cardiometabolic tissue rather than a static marker of visceral adiposity. The consistent reduction of EAT across diverse interventions supports its potential role as a therapeutic target in cardiometabolic disease.

Second, the modest association between BMI and EAT change suggests that systemic weight loss alone may not fully capture therapeutic effects on cardiac adiposity. This is particularly relevant for clinical trials and observational studies that rely on BMI as a surrogate endpoint.

Third, the observed heterogeneity across interventions suggests that therapeutic modality may influence cardiac fat remodeling independently of weight loss. This raises the possibility that EAT could serve as a complementary imaging biomarker for evaluating cardiometabolic interventions beyond traditional anthropometric measures.

Finally, these findings support the rationale for future interventional studies specifically designed to assess differential effects on EAT, including head-to-head comparisons of pharmacological agents and mechanistic studies investigating tissue-specific responses.

### Strengths and limitations

This study has several strengths, including comprehensive coverage of pharmacological and lifestyle interventions, the use of multilevel meta-regression to account for within-study dependencies, and sensitivity analyses addressing key modeling assumptions. In addition, the inclusion of both absolute and BMI-adjusted analyses provides complementary perspectives on EAT responsiveness.

Several limitations should be acknowledged. First, because analyses were based on aggregate study-level data, associations between BMI and EAT changes may be subject to ecological bias and cross-study confounding and should not be interpreted as individual-level relationships. Second, substantial heterogeneity was observed across studies, reflecting differences in population characteristics, intervention intensity, duration, and study design. In addition, the multivariable meta-regression included several intervention categories relative to the available number of intervention arms. Consequently, moderator estimates for intervention classes represented by relatively few studies may be imprecise and should be interpreted as exploratory.

Third, imaging modalities used to assess EAT were heterogeneous, with echocardiography measuring linear thickness and CT/MRI providing volumetric estimates. Although related, these metrics are not fully interchangeable, which may contribute to measurement variability.

Fourth, the assumed within-study correlation between baseline and follow-up measurements introduces uncertainty, although sensitivity analyses across plausible values did not materially alter findings.

Fifth, lack of individual participant data precluded adjustment for important covariates such as sex, visceral fat distribution, medication adherence, and metabolic phenotype.

Sixth, because analyses were performed at the study level, associations between BMI change and EAT change are susceptible to ecological bias. Consequently, intervention-specific deviations from the overall BMI–EAT relationship may reflect differences in patient characteristics, disease phenotypes, baseline metabolic status, or study design rather than true intervention-specific biological effects. Individual participant data meta-analyses will be required to disentangle these influences.

Seventh, although several pooled effects reached statistical significance, the clinical significance of standardized effect sizes remains uncertain. SMCR values facilitate comparison across studies using different imaging modalities but do not directly translate into clinically meaningful thresholds of EAT reduction. Future studies should determine the magnitude of EAT change associated with improvements in cardiovascular outcomes.

Finally, the residual-based estimates of “BMI-adjusted EAT change” are derived from model residuals and should be interpreted as descriptive deviations rather than causal or mechanistic effects.

Substantial between-study heterogeneity was observed, which may influence the performance of funnel plot–based methods. Although Egger’s regression test suggested possible small-study effects, trim-and-fill analysis did not identify missing studies and yielded an identical pooled estimate, supporting the robustness of the primary findings. Nevertheless, the possibility of residual publication bias cannot be completely excluded.

The present analysis focused exclusively on quantitative measures of EAT. Emerging evidence suggests that EAT composition, inflammatory activity, radiomic characteristics, and tissue density may provide complementary information beyond volume alone [5, 15, 16]. Future studies should investigate whether interventions differentially influence qualitative aspects of EAT biology and whether such changes are more closely linked to cardiovascular outcomes.

## Conclusions

Epicardial adipose tissue is a modifiable cardiometabolic fat depot whose response to intervention is only partially explained by systemic weight loss. Substantial heterogeneity exists across intervention classes, suggesting that EAT is influenced by both systemic metabolic changes and additional intervention-specific factors. These findings support the concept of EAT as a distinct and potentially targetable component of cardiometabolic disease, warranting further mechanistic and interventional investigation.

## Supplementary Information

Below is the link to the electronic supplementary material.

Supplementary Material 1

## Data Availability

The datasets used and/or analysed during the current study are available from the corresponding author on reasonable request.

## Abbreviations

AF: Atrial fibrillation
AMPK: AMP-activated protein kinase
BMI: Body mass index
CAD: Coronary artery disease
CENTRAL: Cochrane central register of controlled trials
CI: Confidence interval
CT: Computed tomography
DPP-4: Dipeptidyl peptidase-4
EAT: Epicardial adipose tissue
Echo: Echocardiography
EPA: Eicosapentaenoic acid
GLP-1 RA: Glucagon-like peptide-1 receptor agonist
GRADE: Grading of recommendations assessment, development and evaluation
HFpEF: Heart failure with preserved ejection fraction
HFrEF: Heart failure with reduced ejection fraction
I²: Higgins inconsistency statistic
IQR: Interquartile range
MRI: Magnetic resonance imaging
PRISMA: Preferred reporting items for systematic reviews and meta-analyses
PROSPERO: International prospective register of systematic reviews
QM: Test of moderators in meta-regression
REML: Restricted maximum likelihood
ROBINS-I: Risk of bias in non-randomized studies - of interventions
SD: Standard deviation
SE: Standard error
SGLT-2i: Sodium-glucose cotransporter-2 inhibitor
SMCR: Standardized mean change with repeated measures
TZD: Thiazolidinedione
τ²: Between-study variance
