# Supplementary material for: Association between body mass index reduction and epicardial adipose tissue changes across pharmacological and lifestyle interventions: a multilevel meta-regression analysis
Source: Cardiovasc Diabetol. 2026 Jul 19;25:304. doi: 10.1186/s12933-026-03299-6 (PMC13670704; doi:10.1186/s12933-026-03299-6)
Supplement: Supplementary file 1 [file 12933_2026_3299_MOESM1_ESM.docx]

## **Supplementary Data**

**1. Search Strategy**

A systematic literature search was performed in **MEDLINE (via PubMed), Embase, and the Cochrane Central Register of Controlled Trials (CENTRAL)** from database inception to 22 April 2026. In addition, clinical trial registries (**ClinicalTrials.gov** and the **WHO International Clinical Trials Registry Platform**) and reference lists of relevant articles were manually screened to identify additional studies.

The search strategy combined controlled vocabulary (MeSH and Emtree terms) and free-text terms related to epicardial adipose tissue and cardiometabolic interventions. Key terms included *“epicardial adipose tissue,” “epicardial fat,” “cardiac adipose tissue”* in combination with *“body mass index,” “BMI,” “weight”* and intervention-related terms (*“exercise,” “diet,” “bariatric surgery,” “GLP-1 receptor agonist,” “SGLT2 inhibitor,” “metformin,” “statin,” “thiazolidinedione,” “DPP-4 inhibitor,” “omega-3 fatty acids”*).

Search strategies were adapted for each database. No date restrictions were applied. Searches were limited to human studies. No language restrictions were applied at the search stage; however, only studies with extractable data available in English were included. A representative PubMed search strategy is provided below:

("epicardial adipose tissue" OR "epicardial fat" OR "cardiac adipose tissue") 
AND ("body mass index" OR BMI OR weight) 
AND (exercise OR diet OR "bariatric surgery" OR "GLP-1 receptor agonist" OR "SGLT2 inhibitor" OR metformin OR statin OR thiazolidinedione OR "DPP-4 inhibitor" OR "omega-3 fatty acids")

***Study Selection***

All records were imported into reference management software, and duplicates were removed prior to screening. Titles and abstracts were screened independently by **two reviewers**, followed by full-text assessment of potentially eligible studies.

Full-text articles were independently reviewed by the same two reviewers against predefined inclusion and exclusion criteria. Disagreements were resolved by consensus. A third reviewer was not required.

Eligibility required reporting of pre- and post-intervention mean values with corresponding measures of variance for both epicardial adipose tissue and body mass index.

No automation tools were used in the study selection process.

***Data Extraction***

Data were extracted independently by **two reviewers** using a standardized data collection form. Extracted variables included study design, population characteristics, intervention details, imaging modality, and outcome measures (EAT and BMI at baseline and follow-up).

Extracted data were cross-checked, and discrepancies were resolved by consensus. Where necessary, data were derived from reported statistics (e.g., standard errors or confidence intervals). Study authors were not contacted for additional information.

No automated tools were used for data extraction. Data preprocessing and verification were conducted using R prior to analysis.

**Table S1. Multilevel meta-analysis of intervention effects with variance decomposition across hierarchical study levels**

| **Outcome** | **SMCR** | **SE** | **CI** | **z** | **p** | **τ^2^ Study** | **τ^2^ Arm** | **Between Study (%)** | **Between Arm (%)** | **Within (%)** |
| --- | --- | --- | --- | --- | --- | --- | --- | --- | --- | --- |
| EAT | -0.561 | 0.060 | -0.681 to -0.442 | -9.22 | <0.001 | 0.018 | 0.116 | 9.42% | 62.6% | 27.9% |
| BMI | -0.781 | 0.145 | -1.07 to -0.496 | -5.38 | <0.001 | 0.829 | <0.001 | 93.3% | <0.001% | 6.74% |

Standardized mean change with repeated measures (SMCR) is presented for each outcome, along with standard errors, confidence intervals, and tests of significance. Variance components (τ²) are estimated from a multilevel random-effects model and represent heterogeneity at the between-study and between-arm levels. Percentage values indicate the proportion of total variance attributable to each level (between-study, between-arm, and within-study/residual). SMCR = standardized mean change with repeated measures; SE = standard error; CI = confidence interval; z = test statistic; p = p-value; τ² = variance component; EAT = epicardial adipose tissue; BMI = body mass index.

### **2. Sensitivity and Robustness Analyses**

To evaluate the robustness of pooled estimates to the choice of standardized mean change metric, we compared results derived using SMCC (standardized mean change with correlation correction) and SMCR (standardized mean change using pre-test standardization without explicit correlation correction).

Across all included studies, effect sizes derived from SMCC and SMCR were highly concordant. At the meta-analytic level, differences between methods were small. For epicardial adipose tissue (EAT), the pooled estimate under SMCC was −0.593 (95% CI −0.720 to −0.466), compared with −0.561 (95% CI −0.681 to −0.442) under SMCR, yielding an absolute difference of 0.032 (5.33%). For body mass index (BMI), pooled effects were −0.809 (95% CI −1.10 to −0.520) for SMCC and −0.781 (95% CI −1.07 to −0.496) for SMCR, corresponding to an absolute difference of 0.028 (3.46%) (Table S2).

Variance decomposition was virtually unchanged between approaches. For EAT, heterogeneity remained predominantly attributable to between-arm (within-study) differences, with smaller contributions from within-study and between-study variance. For BMI, variability was almost entirely driven by between-study heterogeneity, with negligible between-arm contribution.

These findings indicate that the principal conclusions were robust to the choice of effect-size metric, with only minor quantitative differences observed between SMCC and SMCR estimates. The small magnitude of differences (<6%) further supports the use of SMCR as the primary effect size metric in the main analyses.

# **Table S2. Sensitivity analysis comparing standardized mean change estimates using SMCC and SMCR within multilevel meta-analytic models**

| Outcome | SMCC | SMCC CI | SMCC p | SMCR | SMCR CI | SMCR p | Abs Diff | % Change | τ² Study | τ² Arm | Between Study | Between Arm | Within |
| --- | --- | --- | --- | --- | --- | --- | --- | --- | --- | --- | --- | --- | --- |
| EAT | -0.593 | -0.720 to -0.466 | <0.001 | -0.561 | -0.681to -0.442 | <0.001 | 0.0316 | 5.33% | 0.0175 | 0.116 | 9.42% | 62.6% | 27.9% |
| BMI | -0.809 | -1.10 to -0.520 to | <0.001 | -0.781 | -1.07 to -0.496 | <0.001 | 0.0280 | 3.46% | 0.829 | <0.001 | 93.3% | <0.001% | 6.74% |

# Pooled effect sizes for epicardial adipose tissue (EAT) and body mass index (BMI) are presented using two approaches: standardized mean change with correlation correction (SMCC) and standardized mean change using pre-test standardization (SMCR). Confidence intervals and p-values are shown for each method. Absolute difference represents the numerical difference between SMCC and SMCR estimates, and percent change reflects the relative difference between methods. Variance components (τ²) and their proportional contributions (%) are derived from multilevel random-effects models and quantify heterogeneity at the between-study, between-arm, and within-study (residual) levels. SMCC = standardized mean change with correlation correction; SMCR = standardized mean change using pre-test standardization; CI = confidence interval; τ² = variance component; EAT = epicardial adipose tissue; BMI = body mass index

### **3. Model Stability Analyses**

We further assessed the robustness of pooled estimates for epicardial adipose tissue (EAT) and body mass index (BMI) using a series of predefined sensitivity analyses within a multilevel random-effects meta-analytic framework (restricted maximum likelihood estimator).

Analyses included (i) variation in the assumed within-study pre–post correlation (r = 0.3, 0.5, 0.7) for standardized mean change using pre-test standardization (SMCR), and (ii) exclusion of small studies (n < 20).

Across all models, effect sizes remained directionally consistent and of similar magnitude. For EAT, pooled SMCR estimates ranged from −0.560 to −0.563 across correlation assumptions, and for BMI from −0.778 to −0.784. Variation in the assumed correlation had minimal impact on both effect size estimates and variance decomposition.

Exclusion of small studies yielded slightly larger effect sizes (EAT: −0.587; BMI: −0.885), with overlapping confidence intervals compared with the primary analysis.

The hierarchical structure of heterogeneity remained stable across all models. For EAT, variability was consistently driven primarily by between-arm (within-study) differences, whereas for BMI, variability was almost entirely attributable to between-study heterogeneity, with negligible between-arm contribution.

Overall, these findings support the robustness of both pooled estimates and variance structure across a range of plausible analytical assumptions (Table S3).

# **Table S3. Sensitivity analysis of pooled SMCR estimates under varying assumptions within multilevel meta-analytic models**

| Outcome | Model | N studies | N arms | SMCR | SE | CI | z | p | Tau2 Study | Tau2 Arm | Between Study | Between Arm | Within |
| --- | --- | --- | --- | --- | --- | --- | --- | --- | --- | --- | --- | --- | --- |
| EAT | r=0.3 | 42 | 50 | -0.563 | 0.061 | -0.683 to -0.443 | 9.19 | <.001 | 0.0161 | 0.107 | 8.43% | 55.9% | 35.7% |
| BMI | r=0.3 | 42 | 50 | -0.784 | 0.146 | -1.07 to -0.498 | 5.37 | <.001 | 0.823 | <0.001 | 91.5% | <0.001% | 8.49% |
| EAT | r=0.5 | 42 | 50 | -0.561 | 0.061 | -0.681 to -0.442 | 9.22 | <.001 | 0.0175 | 0.116 | 9.42% | 62.6% | 27.9% |
| BMI | r=0.5 | 42 | 50 | -0.781 | 0.145 | -1.07 to -0.496 | 5.38 | <.001 | 0.829 | <0.001 | 93.3% | <0.001% | 6.74% |
| EAT | r=0.7 | 42 | 50 | -0.560 | 0.061 | -0.678 to -0.441 | 9.26 | <.001 | 0.0190 | 0.126 | 10.6% | 69.8% | 19.6% |
| BMI | r=0.7 | 42 | 50 | -0.778 | 0.145 | -1.06 to -0.495 | 5.38 | <.001 | 0.836 | <0.001 | 95.1% | <0.001% | 4.93% |
| EAT | r=0.5 (n≥20) | 34 | 40 | -0.587 | 0.072 | -0.727 to -0.446 | 8.20 | <.001 | 0.0258 | 0.134 | 13% | 67.3% | 19.7% |
| BMI | r=0.5 (n≥20) | 34 | 40 | -0.885 | 0.169 | -1.22 to -0.555 | 5.25 | <.001 | 0.921 | <0.001 | 95% | <0.001% | 4.96% |

# Pooled standardized mean change using pre-test standardization (SMCR) is presented for epicardial adipose tissue (EAT) and body mass index (BMI) under different analytical scenarios, including variation in the assumed within-study pre–post correlation (r = 0.3–0.7) and exclusion of small studies (n < 20). Effect sizes, standard errors, confidence intervals, and tests of significance are shown for each model. Variance components (τ²) and their proportional contributions (%) quantify heterogeneity at the between-study, between-arm, and within-study (residual) levels. SMCR = standardized mean change using pre-test standardization; SE = standard error; CI = confidence interval; τ² = variance component; r = assumed pre–post correlation; EAT = epicardial adipose tissue; BMI = body mass index.

**4. Influence and Outlier Diagnostics**

We performed:

- leave-one-out influence analysis (Figure S1)
- Baujat diagnostic analysis to quantify each study’s contribution to heterogeneity and pooled effect size (Figure S2)

These analyses identified whether individual studies disproportionately influenced pooled estimates or heterogeneity statistics.

Across all sensitivity analyses, pooled effect sizes for both EAT and BMI remained directionally consistent, confirming a stable reduction in cardiometabolic adiposity following interventions.

The primary models demonstrated substantial heterogeneity: EAT: I² ≈ 89% and BMI: I² ≈ 95%, indicating considerable between-study variability. However, leave-one-out analyses (Figure S1) showed no single study materially altered pooled estimates, supporting robustness of the overall findings.

Baujat diagnostics (Figure S2) identified two studies (Kim et al., 2009; Iacobellis et al., 2017) as having disproportionately high influence on both pooled effect size and heterogeneity. Nevertheless, exclusion of these studies did not materially alter the magnitude or statistical significance of the BMI–EAT association in multivariable meta-regression models

These studies contributed to the upper-right quadrant of the Baujat plot, indicating simultaneous high contribution to Q-statistic (heterogeneity) and high influence on pooled estimate. However, exclusion sensitivity analyses confirmed that removal of these studies did not materially alter overall conclusions.

**Figure S1. Leave-one-out sensitivity analysis**

Leave-one-out influence diagnostics showing the stability of pooled effect sizes for EAT reduction. Removal of individual studies did not materially alter the overall effect estimate, indicating robustness of the meta-analytic results.

**Figure S2. Baujat plot of study contributions to heterogeneity and pooled effect in the meta-analysis of EAT change**

This Baujat plot displays each study’s contribution to the overall heterogeneity and its influence on the pooled effect size (standardized mean change in epicardial adipose tissue, SMCC). The x-axis represents each study’s contribution to the total heterogeneity (Q statistic contribution), while the y-axis represents its influence on the overall pooled effect, derived from influence diagnostics (DFBETAS for the intercept). Each point corresponds to one study (n = 50). Grey points indicate all included studies, while red points highlight prespecified outlying studies (Kim *et al.*, 2009; Iacobellis *et al.*, 2017)), which were selected for focused interpretation due to their disproportionate influence.

Studies located toward the upper-right quadrant contribute more strongly to both heterogeneity and the pooled effect estimate, indicating higher potential influence on the overall results. Conversely, studies closer to the origin contribute minimally to between-study heterogeneity and have limited impact on the pooled effect. The plot complements the random-effects model (REML estimator, τ² = 0.157, I² = 82.6%), supporting substantial between-study variability and identifying influential studies that may drive this heterogeneity.

Two studies (Kim *et al.*, 2009; Iacobellis *et al.*, 2017) demonstrated disproportionately high influence on both heterogeneity and pooled effect size. This was driven by a combination of large standardized effect estimates and, in the case of Iacobellis et al., 2017, particularly low within-study variance resulting in high statistical weight. These studies therefore exerted high leverage on both the Q statistic and the overall random-effects estimate

The pooled effect size remained stable across all sensitivity analyses (SMCC 0.70 in the full model vs 0.68 after excluding both studies), indicating that the overall conclusion of a significant reduction in EAT is robust to removal of influential studies. In contrast, heterogeneity decreased more substantially (I² from 88.9% to 83.2% and τ² from 0.157 to 0.098 after removing both Kim and Iacobellis), confirming that these studies contribute disproportionately to between-study variability rather than driving the overall effect estimate.

**5. Hierarchical multivariable meta-regression**

# **Table S4. EAT–BMI Meta-Regression Coefficients**

| Model | Term | Estimate | SE | CI_low | CI_high | z | p |
| --- | --- | --- | --- | --- | --- | --- | --- |
| Model 1 | intrcpt | 0.253 | 0.194 | -0.127 | 0.634 | 1.305 | 0.192 |
| Model 1 | yi_BMI | 0.221 | 0.086 | 0.052 | 0.391 | 2.558 | 0.011 |
| Model 1 | GroupDiet | 0.595 | 0.236 | 0.133 | 1.058 | 2.524 | 0.012 |
| Model 1 | GroupDPP-4 i | 0.122 | 0.287 | -0.439 | 0.684 | 0.426 | 0.670 |
| Model 1 | GroupExercise | 0.100 | 0.199 | -0.290 | 0.490 | 0.503 | 0.615 |
| Model 1 | GroupGLP-1 RA | 0.562 | 0.198 | 0.174 | 0.950 | 2.836 | 0.005 |
| Model 1 | GroupMetformin | -0.158 | 0.215 | -0.579 | 0.262 | -0.738 | 0.461 |
| Model 1 | GroupOmega-3 FFA | -0.080 | 0.305 | -0.677 | 0.518 | -0.261 | 0.794 |
| Model 1 | GroupSGLT-2 i | 0.180 | 0.221 | -0.254 | 0.614 | 0.814 | 0.415 |
| Model 1 | GroupStatin | -0.097 | 0.276 | -0.637 | 0.443 | -0.351 | 0.725 |
| Model 1 | GroupTZD | 0.081 | 0.443 | -0.788 | 0.949 | 0.182 | 0.856 |
| Model 2 | intrcpt | 0.262 | 0.211 | -0.151 | 0.675 | 1.243 | 0.214 |
| Model 2 | yi_BMI | 0.224 | 0.090 | 0.048 | 0.400 | 2.495 | 0.013 |
| Model 2 | GroupDiet | 0.590 | 0.246 | 0.107 | 1.072 | 2.395 | 0.017 |
| Model 2 | GroupDPP-4 i | 0.122 | 0.291 | -0.449 | 0.692 | 0.419 | 0.675 |
| Model 2 | GroupExercise | 0.096 | 0.205 | -0.306 | 0.498 | 0.469 | 0.639 |
| Model 2 | GroupGLP-1 RA | 0.562 | 0.202 | 0.166 | 0.958 | 2.782 | 0.005 |
| Model 2 | GroupMetformin | -0.163 | 0.218 | -0.591 | 0.265 | -0.747 | 0.455 |
| Model 2 | GroupOmega-3 FFA | -0.056 | 0.357 | -0.756 | 0.644 | -0.157 | 0.875 |
| Model 2 | GroupSGLT-2 i | 0.179 | 0.226 | -0.263 | 0.621 | 0.795 | 0.427 |
| Model 2 | GroupStatin | -0.098 | 0.281 | -0.649 | 0.453 | -0.349 | 0.727 |
| Model 2 | GroupTZD | 0.081 | 0.449 | -0.799 | 0.961 | 0.181 | 0.857 |
| Model 2 | duration | -0.001 | 0.012 | -0.025 | 0.022 | -0.125 | 0.901 |
| Model 3 | intrcpt | 0.191 | 0.222 | -0.245 | 0.628 | 0.860 | 0.390 |
| Model 3 | yi_BMI | 0.187 | 0.086 | 0.019 | 0.355 | 2.176 | 0.030 |
| Model 3 | GroupDiet | 0.661 | 0.237 | 0.195 | 1.126 | 2.783 | 0.005 |
| Model 3 | GroupDPP-4 i | 0.140 | 0.281 | -0.410 | 0.690 | 0.500 | 0.617 |
| Model 3 | GroupExercise | 0.145 | 0.199 | -0.245 | 0.534 | 0.729 | 0.466 |
| Model 3 | GroupGLP-1 RA | 0.577 | 0.197 | 0.190 | 0.963 | 2.922 | 0.003 |
| Model 3 | GroupMetformin | -0.172 | 0.212 | -0.587 | 0.242 | -0.814 | 0.416 |
| Model 3 | GroupOmega-3 FFA | -0.052 | 0.353 | -0.743 | 0.640 | -0.146 | 0.884 |
| Model 3 | GroupSGLT-2 i | 0.232 | 0.217 | -0.194 | 0.657 | 1.067 | 0.286 |
| Model 3 | GroupStatin | -0.140 | 0.260 | -0.649 | 0.370 | -0.537 | 0.591 |
| Model 3 | GroupTZD | 0.249 | 0.439 | -0.610 | 1.109 | 0.568 | 0.570 |
| Model 3 | duration | 0.001 | 0.011 | -0.021 | 0.023 | 0.111 | 0.912 |
| Model 3 | Imaging.methodEcho | 0.171 | 0.166 | -0.155 | 0.497 | 1.028 | 0.304 |
| Model 3 | Imaging.methodMRI | -0.118 | 0.185 | -0.481 | 0.245 | -0.636 | 0.525 |

## **Model fit and heterogeneity**

| Model | Tau2 | AIC | BIC |
| --- | --- | --- | --- |
| Model 1 | 0.068 | 50.313 | 71.940 |
| Model 2 | 0.072 | 52.666 | 75.593 |
| Model 3 | 0.057 | 51.561 | 76.897 |

Across all models, BMI change was positively associated with EAT reduction. In the fully adjusted model, each one-unit increase in BMI effect size was associated with a 0.187-unit increase in EAT effect size (β = 0.187, 95% CI 0.019–0.355; p = 0.030). Intervention duration was not associated with EAT response, and imaging modality did not independently explain residual heterogeneity. Relative to the reference intervention category, dietary interventions and GLP-1 receptor agonists demonstrated larger EAT reductions than predicted by the average BMI–EAT relationship.

These intervention-specific coefficients should be interpreted cautiously. Because analyses were performed at the study level, observed differences between intervention classes may reflect differences in patient populations, baseline risk profiles, study design, or other unmeasured factors rather than true intervention-specific biological effects.

**6. Variance decomposition**

Variance decomposition demonstrated distinct heterogeneity structures for EAT and BMI. For EAT, most variability arose from differences between intervention arms within studies (62.6%), whereas between-study variance accounted for only 9.4% of total heterogeneity. In contrast, BMI variability was overwhelmingly attributable to between-study differences (93.3%), with negligible between-arm contribution. These findings suggest that EAT responses vary substantially across intervention arms even within the same study, whereas BMI responses are predominantly determined by differences between study populations and study-level characteristics.

We decomposed total variance into: Level 1: sampling variance, level 2: within-study variance and level 3: between-study variance to quantify the hierarchical structure of heterogeneity (Figure S4).

Variance partitioning demonstrated that between-study variance (Level 3) was the dominant component for BMI, while within-study variance was the dominant component for EAT (Fig. S4).

**Figure S4. Variance decomposition for EAT and BMI response (three-level model)**

Decomposition of total variance into sampling variance (Level 1), within-study variance (Level 2), and between-study variance (Level 3). Between-study heterogeneity accounted for the largest proportion of total variance for BMI, while within study variance for EAT.

This confirms that heterogeneity is primarily driven by true clinical and methodological differences between studies rather than random sampling error.

**7. Publication bias**

**Figure S5. Funnel plot and trim-and-fill analysis for publication bias assessment.**

Funnel plot of study-level standardized mean change with raw score standardization (SMCR) estimates for epicardial adipose tissue (EAT). Egger's regression test suggested possible funnel plot asymmetry (z = 2.05, p = 0.040). Duval and Tweedie's trim-and-fill procedure did not identify potentially missing studies (k0 = 0), and the pooled effect estimate remained unchanged after correction, indicating that publication bias is unlikely to materially affect the overall findings.

**Table S5. Assessment of publication bias.**

| **Analysis** | **Result** |
| --- | --- |
| Egger's regression test | z = 2.05, p = 0.040 |
| Number of imputed studies (trim-and-fill) | 0 |
| Original pooled effect (SMCR) | -0.584 (95% CI −0.705 to −0.464) |
| Trim-and-fill adjusted effect (SMCR) | -0.584 (95% CI −0.705 to −0.464) |

**Table S6. Publication bias results**

| **Metric** | **Value** |
| --- | --- |
| Egger z | 2.05 |
| Egger p | 0.040 |
| Missing studies (trim-fill) | 0 |
| Original estimate | -0.584 |
| Adjusted estimate | -0.584 |

Results of publication bias analyses performed using study-level effect estimates. Although Egger's regression test indicated possible funnel plot asymmetry, trim-and-fill analysis identified no potentially missing studies and did not alter the pooled effect estimate.

**GRADE evidence profile**

**Table S7. GRADE evidence profile for primary outcomes and exploratory analyses**

| **Outcome** | **No. studies (arms)** | **Study design** | **Risk of bias** | **Inconsistency** | **Indirectness** | **Imprecision** | **Publication bias** | **Overall certainty** |
| --- | --- | --- | --- | --- | --- | --- | --- | --- |
| Reduction in EAT following intervention (overall pooled effect) | 42 studies (50 arms) | Predominantly single-arm interventional studies, some randomized controlled trials | Serious | Serious | Not serious | Serious | Undetected* | LOW |
| Association between BMI change and EAT change (multilevel meta-regression) | 42 studies (50 arms) | Study-level observational association derived from intervention studies | Serious | Serious | Serious | Serious | Undetected* | VERY LOW |
| Intervention-class differences in EAT response | 42 studies (50 arms) | Exploratory study-level meta-regression | Serious | Serious | Serious | Serious | Undetected* | VERY LOW |
| Dietary interventions and greater-than-predicted EAT reduction | 3 studies (3 arms)** | Exploratory subgroup/meta-regression finding | Serious | Serious | Serious | Very serious | Undetected* | VERY LOW |
| GLP-1 receptor agonists and greater-than-predicted EAT reduction | 7 studies (8 arms)** | Exploratory subgroup/meta-regression finding | Serious | Serious | Serious | Very serious | Undetected* | VERY LOW |

*Egger’s regression suggested possible small-study effects; however, trim-and-fill analysis did not identify missing studies and did not materially alter pooled estimates.

GRADE assessments were adapted for meta-analytic evidence derived predominantly from single-arm intervention studies. Certainty ratings were downgraded for:

• Risk of bias: frequent inclusion of non-randomized studies, single-arm designs, and small sample sizes.

• Inconsistency: substantial residual heterogeneity across studies (I² generally >70%).

• Indirectness: heterogeneity in populations, interventions, imaging modalities, and the study-level nature of meta-regression analyses.

• Imprecision: limited sample sizes within several intervention classes and wide confidence intervals for subgroup estimates.

• Publication bias: exploratory analyses suggested possible small-study effects, although trim-and-fill analyses did not materially alter pooled estimates.

Intervention-specific findings derived from meta-regression analyses were considered hypothesis-generating and were downgraded further because of ecological bias, residual confounding, and limited numbers of studies within several intervention categories.

**References**

Iacobellis G, Mohseni M, Bianco SD, Banga PK (2017). Liraglutide causes large and rapid epicardial fat reduction. *Obesity* 25**:** 311-316.

Kim MK, Tanaka K, Kim MJ, Matuso T, Endo T, Tomita T*, et al.* (2009). Comparison of epicardial, abdominal and regional fat compartments in response to weight loss. *Nutrition, metabolism, and cardiovascular diseases : NMCD* 19**:** 760-766.
